# Fusion Model Using Resting Neurophysiological Data to Help Mass Screening of Methamphetamine Use Disorder

**DOI:** 10.1109/JTEHM.2024.3522356

**Published:** 2024-12-25

**Authors:** Chun-Chuan Chen, Meng-Chang Tsai, Eric Hsiao-Kuang Wu, Shao-Rong Sheng, Jia-Jeng Lee, Yung-En Lu, Shih-Ching Yeh

**Affiliations:** Department of Biomedical Sciences and EngineeringNational Central University 34911 Taoyuan 320 Taiwan; Department of PsychiatryKaohsiung Chang Gung Memorial Hospital Kaohsiung 833 Taiwan; School of MedicineChang Gung University 56081 Taoyuan 320 Taiwan; Computer Science and Information Engineering DepartmentNational Central University 34911 Taoyuan 320 Taiwan; Department of Computer Science and Information EngineeringNational Cheng Kung University 34912 Tainan 701 Taiwan

**Keywords:** Methamphetamine (MA), bio-signal, heart rate variability (HRV), electrocardiography (ECG), electroencephalography (EEG), galvanic skin response (GSR), data fusion, multimodal data, virtual reality (VR), machine learning

## Abstract

Methamphetamine use disorder (MUD) is a substance use disorder. Because MUD has become more prevalent due to the COVID-19 pandemic, alternative ways to help the efficiency of mass screening of MUD are important. Previous studies used electroencephalogram (EEG), heart rate variability (HRV), and galvanic skin response (GSR) aberrations during the virtual reality (VR) induction of drug craving to accurately separate patients with MUD from the healthy controls. However, whether these abnormalities present without induction of drug-cue reactivity to enable separation between patients and healthy subjects remains unclear. Here, we propose a clinically comparable intelligent system using the fusion of 5–channel EEG, HRV, and GSR data during resting state to aid in detecting MUD. Forty-six patients with MUD and 26 healthy controls were recruited and machine learning methods were employed to systematically compare the classification results of different fusion models. The analytic results revealed that the fusion of HRV and GSR features leads to the most accurate separation rate of 79%. The use of EEG, HRV, and GSR features provides more robust information, leading to relatively similar and enhanced accuracy across different classifiers. In conclusion, we demonstrated that a clinically applicable intelligent system using resting-state EEG, ECG, and GSR features without the induction of drug cue reactivity enhances the detection of MUD. This system is easy to implement in the clinical setting and can save a lot of time on setting up and experimenting while maintaining excellent accuracy to assist in mass screening of MUD.

## I. Introduction

Methamphetamine (MA) is one of the most-used addictive psychostimulants worldwide*, becoming even more prevalent due to the COVID-19 pandemic [1]. Abuse of methamphetamine can permanently damage brain cells [2] and lead to methamphetamine use disorder (MUD). Patients with MUD suffer from significant neurological and psychiatric abnormalities (see [3], [4] for review) and even early death [5]. Currently, clinicians use the Diagnostic and Statistical Manual of Mental Disorders – 5th Edition (DSM-5) to diagnose methamphetamine-related mental health disorders. The DSM-5 has some limitations for diagnosis, such as the time required for its application and potential cultural or contextual factors that might introduce biases or inaccuracies. Using neuro-sensing devices, it was reported that patients with MUD exhibit significant electroencephalogram (EEG), heart rate variability (HRV) and galvanic skin response (GSR) abnormalities during VR induction of drug craving [6], [7], [8], [9], [10]. The neurophysiological abnormalities that occur during or after drug-cue induction provide data to help accurately separate patients with MUD from the healthy controls [6], [7], [8] and can serve as potential neuro-biomarkers for MUD diagnosis. However, the separation rate decreased when distinguishing patients with MUD from healthy subjects using only a few EEG features without VR induction of drug-cue reactivity [6]. Additionally, whether the HRV and GSR abnormalities seen in MUD patients are irrelevant to drug cues remains unclear. In other words, it has not yet been tested whether resting-state HRV and GSR features can differentiate patients with MUD from healthy subjects. Given that patients with MUD exhibit significant EEG, HRV and GSR aberrations during and after the VR induction of drug craving, in this study, we hypothesize that the combination of these neurophysiological features using resting-state data without inducing drug cue reactivity can aid in accurately separating patients with MUD from healthy controls. Fusion model is preferred as abnormalities in each single modality under resting states may reflect different diseases, for instance, arrhythmia (HRV alteration), palmar hyperhidrosis (higher GSR features), or depression (EEG changes). Using resting-state data is crucial because it avoids the risk of triggering drug cravings, especially when the focus is on screening rather than treatment. Additionally, this approach significantly reduces the time required for setup and examination while maintaining high accuracy, making it ideal for mass screening. Finally, the system is versatile and can be easily implemented in various environments, including community settings and hospitals.

## II. Related Work

Patients with MUD display several clinical neurological and cognitive impairments, including mood disturbances, persistent cravings, psychosis, and deficits in attention, language and verbal fluency, verbal learning and memory, and working memory (see [3], [4] for review). To study the underlying mechanisms of MUD, drug cue reactivity, including mental (e.g., craving and attention bias), psychophysiological (e.g., heart rate, skin conductance, and brain activity), and behavioral (e.g., drug-seeking) responses, is evoked experimentally using visual, auditory, or olfactory stimuli associated with previous drug use [11], [12]. Drug cue reactivity is recorded using neurophysiological sensing devices for EEG, electrocardiography (ECG), and GSR. Specifically, in substance abuse disorders, EEG features can serve as the endophenotypes to discover the biological underpinnings of neurocognitive and neurophysiological impairments [13]. Previous studies have reported that patients with MUD exhibited significant EEG abnormalities in $\delta $, $\ominus $, $\alpha $, and $\gamma $ power changes during or after induction of drug craving [6], [7], [10]. Furthermore, these EEG features have been used to separate patients with MUD from healthy subjects with an accuracy greater than 86% [6], [7]. In 2019, Khajehpour et al.used brain functional connectivity features during resting-state and SVM classifier to aid in MUD classification with very high accuracy (93%) [14]. In 2020, Chen et al.reported that patients with MUD exhibited an abnormal resting-state EEG microstate and disrupted resting network dynamics and cognitive functions [15]. Both of the above-mentioned resting-state studies used data from a high-density EEG array (>62 channels). However, separation accuracy decreased considerably when only using EEG features from five channels without VR induction of drug cue reactivity (i.e., before VR) [6]. One possible explanation is that using only a few EEG channels may not provide adequate information for separating the two groups.

Heart rate is a key vital sign of normal physiological condition. Heart rate variability (i.e. HRV) measures the beat-to-beat variation in either heart rate or the duration of the R-R interval extracted from ECG signal and has become an important clinical and research character. HRV analysis estimates the cardiac autonomic modulation of the autonomic nervous system (ANS) and is used in studying different diseases [16], [17], [18], [19]. In patients with MUD, cardiac complications, particularly hypertension, aortic dissection, acute coronary syndromes, and pulmonary arterial hypertension, are often seen as MA causes sympathomimetic amines and affects the endogenous agonists of the sympathetic nervous system, resulting in increases in heart rate, force of cardiac contraction, and blood pressure [20], [21]. Yanik et al. showed that the drug for the treatment of pulmonary arterial hypertension can modulate the P wave, T wave and QRS complex in ECG [22]. It was reported that patients with MUD differ significantly from healthy controls in the changes of HRV indexes of time, frequency and nonlinear domains [8], [9], [23]. These HRV changes positively correlated with the level of MA craving in patients with MUD [24].

Galvanic skin response (GSR) measures changes in the electrical characteristics of skin that reflect the activity of the autonomic nervous system. Greater arousal usually leads to higher activity within the autonomic nervous system’s sympathetic branch and enhanced sweat gland activity, which in turn increases skin conductance [25]. Ding et al. used EEG and GSR features to separate patients with MUD from healthy subjects with an accuracy of 86% [6], [7]. Kim et al. used PPG, GSR and EOG data to detect craving for gaming by Support Vector Machine (SVM) model using feature level fusion [26].

In summary, MA abuse leads to abnormalities in the central nervous system which are reflected in EEG, as well as in the autonomic nervous system, altering HRV and GSR. Therefore, it is reasonable to assume that multimodal data comprising EEG, ECG and HRV data may increase separation accuracy of patients with MUD from healthy subjects.

Indeed, the aim of data fusion is to use multiple data sets collected simultaneously from different devices to improve the performance of certain applied processes [27]. There are two main methods for data fusion: feature level fusion and decision level fusion. Feature level fusion, also known as early fusion, is used in a very intuitive way, which is to directly concatenate all inputs in a series to form a longer one-dimensional array. The goal is to find the relevance of features between different datasets to help the classification. The other fusion model is decision level fusion, also known as late fusion. In this fusion model, each dataset undergoes individual training and then the results of each data-specific model are used with different weights to determine the final result. Cai et al. used feature level fusion approaches for three types of features to improve the performance of their depression recognition model with the highest classification accuracy of 86.98% [28]. The decision level fusion method has been used to enhance the model performance of multimodal data such as RB, PPG and FTT [29]. Regarding MUD, previous studies have successfully used the EEG and GSR features [7] or GSR and HRV features to separate patients with MUD from healthy controls under the condition of drug cue exposure [6], [7], [8]. In this study, we aim to test all possible combinations of features extracted from the resting-state EEG, HRV and GSR without any induction of drug cue reactivity.

## III. Methodologies

### A. Experiment

#### 1) Participants

Forty six patients with MUD were recruited from Chang Gung Memorial Hospital to participate in our study. Some of the data have been used in our previous studies [6], [8]. The inclusion criteria were as follows: (1) Aged between 20 and 65 years old; (2) Diagnosed with MUD according to the DSM-5; (3) Without history of major mental illness; (4) Without physical disease or the physical disease must be stable and under control; and (5) Without heart disease. The diagnosis of all patients with MUD was performed by a psychiatrist certified by the board of directors.

Twenty six healthy subjects between the ages of 20 and 65, and without mental, physical or heart disease, were recruited as the control group for comparison. No subject in the control group was taking any prescription medicine.

The study was performed at Kaohsiung Chang Gung Memorial Hospital and was approved by the Institutional Review Board of Kaohsiung Chang Gung Memorial Hospital (201901912A3) in accordance with the Declaration of Helsinki and Good Clinical Practice guidelines. All participants signed an informed consent after receiving an explanation of the study.

#### 2) Resting-State Virtual Reality Scenario and System

In this study, we used a VR system integrated with EEG, ECG and GSR using Unity3D®. This integrated VR system was used in our previous studies [6], [8]. In this study, the participant was seated comfortably with the VR headset on and was instructed to keep still for five minutes with their eyes open. A steady blue sky scene was presented through the VR headset for subjects to passively view for five minutes. The neurophysiological data from EEG, ECG and GSR were measured with the sample rate of 500 Hz, 2048 Hz and 256Hz, respectively.

### B. Data Process

#### 1) EEG Features

Throughout the experiment, EEG data from five channels (C3, C4, Cz, Pz and FCz) were recorded for five minutes. These data were bandpass filtered (2-56 Hz) and transformed into the time-frequency domain from 4 to 48Hz offline using SPM12 (Wellcome Trust Centre for Neuroimaging, https://www.fil.ion.ucl.ac.uk/spm/). The spectral densities at each channel were divided into four frequency bands of interest, including the $\ominus $ (4-7 Hz), $\alpha $ (8-14 Hz), $\beta $ (15-24 Hz) and $\gamma $ (25-48 Hz) bands and averaged across frequencies within the prespecified frequency bands of interest. The resulting first-minute averaged data of each subject were used as the individual baseline. Finally, we obtained 20 EEG frequency features (4 bands x 5 channels) for each subject.

In addition, we directly extracted nine features using time domain data of each channel, including katz fractal dimension, Approximate Entropy, Sample entropy, Permutation entropy, Singular value decomposition entropy, Higuchi fractal dimension, Hjorth mobility, Hjorth complexity and Detrended fluctuation analysis. This results in 45 EEG time domain features. Finally, functional connectivity features during resting-state EEG among the five channels were also computed for comparison [14].

#### 2) HRV and GSR Features

Three-lead ECG electrodes placed in right arm, left arm and left leg were used to measure heart rate and rhythm. The raw ECG data were first down-sampled to 256 Hz and filtered with a band-stop filter of 60 Hz to remove the power line artifacts. Thirty seven HRV features were derived from the interval between two heartbeats, i.e., R-R interval, using pyHRV package, an opensourece python toolbox for HRV (https://pyhrv.readthedocs.io/). These HRV indexes contained 18 time domain- related features, 14 frequency-related features, and 5 nonlinear parameters. GSR was measured by attaching the electrodes to the index finger, where sweat gland density is high. GSR features were acquired by the Procomp5 Infiniti System device® with a sampling rate of 256Hz. Ten GSR features were calculated using Biosppy, an open source python toolbox for biosignal processing (https://biosppy.readthedocs). Supplementary Table 1 lists the descriptions of HRV and GSR features.

**TABLE 1:** Demographic Characteristics

Variable	Patients ( $n=46$)	Controls ( $n=26$)	Significance (P-value)
Age, years	39.0±8.7	34.0±10.2	0.021*
Male	36	16	
BMI	26.1	24.7	0.24

### C. Statistical Analysis

Welch’s t-tests (i.e. Students’ *t*-test assuming unequal variances to effectively account for the sample size imbalance) were used to test for any differences between the two groups. The significant level was set at $p< 0.05$ after correction and the reported p values were set as $p=0.05$ if no longer significant after correction.

### D. Machine Learning Method

All extracted features were used as attributes for several machine learning methods, including decision tree (DT), random forest (RF), logistic regression (LR), Gaussian process (GP), K-nearest neighbor (KNN), ada boost (AD), gradient boost (GB), and multilayer perceptron (MLP) for two-class classification. The aim was to differentiate the datasets between the control and patient groups during resting states. This aim was accomplished in two steps. First, data partitioning: To address the data imbalance, we reserved 20 patient samples as an independent test set, leaving 26 patient samples and 26 control samples for training. Second, we applied five-fold cross-validation to assess detection accuracy. In each fold, a subset of the training data (5 patients and 5 controls) was randomly chosen for testing, and we calculated the mean accuracy for the cycle. Additionally, the pre-reserved 20 patient samples were also used to further validate the model’s construct. This two-step process was repeated 100 times, each time with varied training data, to simulate random sampling effects. Finally, we averaged the accuracies from all iterations for overall detection accuracy and construct validation.

### E. Fusion Model

Having obtained the features from different modalities, we implemented two fusion models: early and late fusion. In early fusion, EEG, HRV and GSR were concatenated into one feature set for training the predictive model. Because dataset value ranges differ, we employed the min-max normalization method to rescale the features. In late fusion, the different features were combined after model building using individual datasets.

## IV. Results

### A. Demographic Characteristics

Table 1 presents statistical results of the demographic characteristics of patients with MUD and healthy subjects. The mean age of the healthy group was significantly younger in this study (*p = 0.021*), possibly due to a smaller sample size. The two groups exhibited similar body mass index.

### B. Modality-Specific Classification Results

We first examined the importance of each modality by only using modality-specific data. As EEG are rich and complex in information, we tested whether different EEG features can significantly change classification results. Table 2 lists the classification results using different EEG features. It can be seen that the time-domain EEG features have the best performance of 0.68 with feature selection and RF classifier. The frequency-related and functional connectivity network features have a similar performance, ranging from 0.46 to 0.62 with and without feature selection.

**TABLE 2:** EEG Features and Classification Results

	Frequency-related features (20 features)				Time domain features (45 features)				Functional connectivity features (15 features)			
Classifier	Accuracy	Precision	Recall	F1-Score	Accuracy	Precision	Recall	F1-Score	Accuracy	Precision	Recall	F1-Score
No Feature Selection												
DT	0.55	0.55	0.58	0.54	0.60	0.64	0.52	0.55	0.58	0.63	0.51	0.53
RF	0.61	0.61	0.63	0.60	0.63	0.65	0.62	0.61	0.51	0.51	0.52	0.50
LR	0.58	0.58	0.62	0.59	0.65	0.64	0.73	0.67	0.60	0.63	0.50	0.53
GP	0.52	0.52	0.56	0.52	0.61	0.62	0.66	0.62	0.47	0.43	0.26	0.30
KNN	0.60	0.59	0.75	0.65	0.65	0.66	0.64	0.64	0.56	0.57	0.52	0.53
AD	0.59	0.59	0.62	0.59	0.62	0.64	0.60	0.60	0.54	0.54	0.58	0.55
GB	0.55	0.56	0.58	0.55	0.54	0.54	0.55	0.53	0.54	0.54	0.59	0.55
MLP	0.61	0.62	0.61	0.59	0.65	0.80	0.40	0.51	0.50	0.00	0.00	0.00
Feature selection from Decision Tree												
DT	0.62	0.64	0.55	0.57	0.60	0.62	0.56	0.55	0.53	0.51	0.56	0.52
RF	0.59	0.60	0.60	0.58	0.63	0.65	0.62	0.61	0.51	0.50	0.50	0.48
LR	0.58	0.58	0.59	0.57	0.64	0.64	0.71	0.66	0.59	0.64	0.43	0.48
GP	0.51	0.51	0.53	0.51	0.57	0.58	0.58	0.56	0.46	0.38	0.22	0.27
KNN	0.56	0.55	0.69	0.60	0.58	0.61	0.52	0.53	0.54	0.56	0.37	0.43
AD	0.59	0.60	0.59	0.58	0.63	0.65	0.62	0.61	0.52	0.52	0.54	0.51
GB	0.58	0.58	0.60	0.57	0.55	0.56	0.56	0.54	0.52	0.52	0.55	0.52
MLP	0.57	0.65	0.24	0.39	0.68	0.68	0.75	0.70	0.61	0.63	0.59	0.59

Then we compared the performance of modality-specific features in Table 3. Among the modalities, HRV features lead to the greater separation accuracy, when compared with EEG and GSR features. The highest accuracy of all was 0.78 when using HRV features and MLP classifier with feature selection. The accuracy ranges across classifiers were from 0.50 to 0.78 for HRV, 0.58 and 0.67 for EEG features, and 0.59 and 0.67 for GSR (Table 3). It should be noted that the frequency-related EEG features alone performed better (best accuracy of 0.62) than the combination of all three EEG feature types (best accuracy of 0.61).

**TABLE 3:** Modality-Specific Classification Results

	EEG features (all)				HRV				GSR			
Classifier	Accuracy	Precision	Recall	F1-Score	Accuracy	Precision	Recall	F1-Score	Accuracy	Precision	Recall	F1-Score
No Feature Selection												
DT	0.67	0.70	0.67	0.65	0.71	0.76	0.68	0.69	0.62	0.65	0.60	0.60
RF	0.66	0.68	0.64	0.64	0.74	0.78	0.72	0.73	0.62	0.62	0.66	0.62
LR	0.63	0.64	0.67	0.63	0.77	0.78	0.79	0.78	0.67	0.66	0.75	0.69
GP	0.62	0.62	0.70	0.64	0.71	0.74	0.70	0.70	0.66	0.65	0.77	0.69
KNN	0.62	0.60	0.82	0.68	0.73	0.80	0.64	0.68	0.63	0.66	0.60	0.61
AD	0.66	0.69	0.63	0.64	0.73	0.75	0.72	0.72	0.63	0.63	0.69	0.64
GB	0.58	0.59	0.59	0.57	0.68	0.69	0.68	0.67	0.59	0.59	0.65	0.60
MLP	0.61	0.57	0.95	0.71	0.50	0.50	0.99	0.67	0.65	0.68	0.65	0.61
Feature selection from Decision Tree												
DT	0.66	0.66	0.71	0.66	0.70	0.79	0.58	0.64	0.60	0.63	0.57	0.57
RF	0.65	0.67	0.64	0.63	0.74	0.78	0.71	0.73	0.62	0.62	0.68	0.63
LR	0.61	0.62	0.64	0.61	0.78	0.80	0.78	0.78	0.64	0.64	0.70	0.66
GP	0.62	0.61	0.71	0.65	0.72	0.75	0.69	0.70	0.63	0.62	0.71	0.65
KNN	0.64	0.63	7.74	0.66	0.75	0.85	0.61	0.69	0.65	0.70	0.59	0.62
AD	0.64	0.67	0.63	0.63	0.74	0.77	0.71	0.72	0.62	0.63	0.69	0.64
GB	0.58	0.59	0.60	0.57	0.68	0.70	0.69	0.67	0.59	0.59	0.66	0.61
MLP	0.67	0.62	0.93	0.74	0.78	0.82	0.79	0.79	0.67	0.65	0.79	0.70

### C. Fusion Model Classification Results

Table 4 lists the fusion results of all possible combinations of features. In the fusion model, we used only the time domain features of EEG because they lead to the best performance (see Table 3). In the early fusion model, the highest accuracy was 0.79 when using HRV and GSR features and LR classifier, followed by EEG, HRV and GSR features with 0.77 accuracy using MLP classifier. For late fusion model, the highest accuracy was 0.71 when using HRV and GSR features and LR classifier, followed by EEG, HRV and GSR features with 0.67 accuracy LR classifier. The fusion of EEG and GSR features was the least accurate, being lower than 0.7 for all classifier. The fusion of EEG and HRV features has a moderate accuracy from 0.59 to 0.74.

**TABLE 4:** Fusion Models and Classification Results

	EEC + HRV				EEG+GSR				HRV+GSR				EEG+HRV+GSR			
Classifier	Accuracy	Precision	Recall	F1-Score	Accuracy	Precision	Recall	F1-Score	Accuracy	Precision	Recall	F1-Score	Accuracy	Precision	Recall	F1-Score
Early Fusion																
DT	0.69	0.71	0.72	0.69	0.63	0.64	0.62	0.61	0.70	0.71	0.72	0.69	0.67	0.70	0.68	0.67
RF	0.71	0.76	0.66	0.68	0.64	0.67	0.62	0.62	0.72	0.76	0.67	0.69	0.70	0.74	0.67	0.68
LR	0.74	6.74	0.78	0.75	0.68	0.69	0.69	0.68	0.79	0.81	0.80	0.79	0.76	0.77	0.78	0.76
GP	0.72	0.73	0.74	0.72	0.60	0.60	0.65	0.61	0.76	0.80	0.73	0.74	0.76	0.76	0.81	0.77
KNN	0.72	6.74	0.74	0.72	0.50	0.57	0.77	0.64	0.71	0.76	0.66	0.69	0.69	0.66	0.84	0.73
AD	0.70	6.73	0.67	0.68	0.64	0.67	0.63	0.62	0.72	0.76	0.79	0.76	0.70	6.73	0.67	0.68
GB	0.65	0.66	0.66	0.64	0.58	0.59	0.58	0.57	0.66	0.69	0.66	0.65	0.63	0.65	0.64	0.62
MLP	0.73	0.69	0.89	0.77	0.70	0.75	0.62	0.66	0.50	0.00	0.00	0.00	0.77	0.78	0.78	0.77
Late Fusion																
DT	0.62	0.62	0.64	0.61	0.58	0.59	0.59	0.56	0.66	0.68	0.66	0.64	0.63	0.64	0.64	0.62
RF	0.66	0.68	0.66	0.65	0.61	0.62	0.64	0.61	0.67	0.69	0.68	0.66	0.65	0.67	0.66	0.65
LR	0.66	0.66	0.69	0.66	0.61	0.61	0.67	0.63	0.71	0.71	0.77	0.72	0.67	0.67	0.72	0.68
GP	0.59	0.61	0.62	0.59	0.58	0.57	0.64	0.59	0.68	0.69	0.74	0.69	0.63	0.64	0.67	0.63
KNN	0.66	0.69	0.70	0.67	0.61	0.62	0.69	0.63	0.68	0.73	0.61	0.64	0.66	0.70	0.66	0.65
AD	0.65	0.66	0.65	0.64	0.60	0.61	0.64	0.61	0.67	0.68	0.70	0.67	0.65	0.66	0.67	0.65
GB	0.60	0.61	0.62	0.59	0.57	0.67	0.60	0.67	0.62	0.63	0.66	0.62	0.60	0.61	0.63	0.60
MLP	0.63	0.62	0.77	0.66	0.62	0.64	0.63	0.60	0.65	0.65	0.79	0.67	0.64	0.64	0.75	0.65

### D. Features Significantly Differ in the Two Groups

**FIGURE 1.**
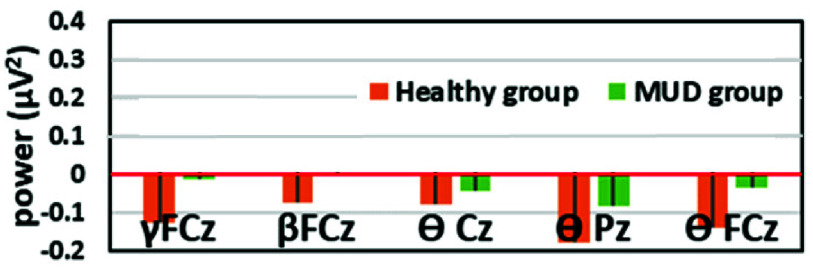
Significant EEG features.

To extract important features, student *t*-test was employed. Figure 1 shows the EEG features that differ significantly between the two groups. Five of 20 frequency-related EEG features significantly differ between the two groups, including theta oscillations at FCz, Cz and Pz and beta and gamma power at FCz. The healthy subjects exhibited greater suppression in all significant frequency bands over the midline channels compared with patients with MUD.

In addition, 2 out of 37 HRV features, nni_diff_max and SDSD were significantly different between the two groups (Table 5). Eight of 10 GSR features were significantly different between the two groups (Table 5). Specifically, these significant GSR features of patient group were always greater in amplitudes when compared with healthy controls, except the standard deviation of the GSR peaks and pulse amplitudes and the average of GSR pulse amplitudes.

**TABLE 5:** Significant HRV and GSR Features

HRV features	P value	HRV features	P value
nni_diff_max	$0.01\ast $	Sdsd	$0.016\ast $
GSR features	P value	GSR features	P value
diff	$< 0.01\ast \ast $	mini_scr	$< 0.01\ast \ast $
startle	$< 0.01\ast \ast $	maxi_scr	$< 0.01\ast \ast $
duration	$< 0.01\ast \ast $	average_scr	$< 0.01\ast \ast $
average_filter	$< 0.01\ast \ast $	std_scr	$0.012\ast $

## V. Discussions and Conclusion

In this study, we proposed a fusion model with EEG, HRV and GSR features during resting states and tested whether this fusion model can help diagnose MUD. We found that the combination of HRV and GSR has the highest accuracy of 79% among all fusion models tested. The fusion of all three feature types has a separation accuracy rate of 77% and a relatively similar classification accuracy among different classifiers. The use of EEG, HRV and GSR features provides more robust information, leading to relatively similar and enhanced accuracy across different classifiers.

### A. The Best Fusion Model of HRV and GSR Features

Both heart rate and sweating are controlled by the autonomic nervous system and modulated by psychological or physiological arousal. Usually, higher activity of the sympathetic branch of the autonomic nervous system enhances heart rate, the activity of sweat glands and skin conductance. Therefore, HRV and GSR can be a measure of emotional and sympathetic responses [30], [31]. Previous studies have successfully used the EEG and GSR features [7] or HRV and GSR features to separate patients with MUD from healthy controls under the condition of drug-cue exposure [6], [7], [8]. In this study, we further found that the fusion model of HRV and GSR features has the most accurate separation rate of 79% when no drug cues were given, suggesting an impaired autonomic nervous system in patients with MUD. Interestingly, the fusion of EEG, HRV and GSR features improved the accuracy (up to 77%) when compared with the results of individual datasets, but the improvement was not as much as expected. One possible reason is that EEG activities exhibit significant between-subject variability that is unrelated to group differences, as the study setting cannot control for individual differences under the resting-state condition. This variability decreases the separation rates of fusion models that include EEG data. Further studies with a larger, more adequate dataset will help to clarify this issue.

### B. Abnormalities in HRV and GSR

Specifically, the irregularity of heart rate measured with max difference in R-R interval was significantly higher in patients with MUD than in healthy subjects in this study. Because atrioventricular arrhythmia is one of the acute cardiovascular impacts of MA use [32], our finding is consistent with the previous report and confirmed the cardiotoxicity seen with MA use.

Regarding GSR abnormalities, GSR activity is thought to reflect the sympathetic arousal associated with cognitive conditions of emotion, cognition, and attention [33]. The neuronal correlates of GSR generation include the ventromedial prefrontal cortex, orbitofrontal cortex, left primary motor cortex, and the anterior/posterior cingulate area, which have been shown to be associated with emotional and motivational behaviors [34]. Specifically, it was shown that the GSR induced by alcohol cues was positively associated with craving and neuronal activities of the prefrontal cortex and thalamus when compared with those induced by neutral cues, although men and women may show different patterns [35]. Because we lacked stimulus cues in this study, the findings of greater GSR features in patients with MUD may reflect the abnormal arousal level and autonomic nervous system even without any sensitive stimulus.

### C. Abnormalities in EEG

In this study, we observed that the resting $\ominus $, $\beta $ and $\gamma $ power significantly differ in the two groups. A previous study reported that the $\ominus $ power in resting-state EEG is significantly higher in impulsive individuals [36]. Furthermore, it was reported that after inducing drug-cue relativities via VR, the power were significantly suppressed in healthy controls but not in patients with MUD, suggesting a higher impulsive level in patients [6]. In this study, we observed significantly higher resting $\ominus $ power in patients with MUD over the midline channels, irrelevant to drug cues. Regarding the $\gamma $ power, one functional role of the $\gamma $ power is related to high-risk choices [37]. Previous studies have reported a reduced $\gamma $ suppression in the frontal areas during or after exposure to drug cues in patients with MUD [6], [10], suggesting that the reduced $\gamma $ suppression may be related to the deficit in impulse control [6]. In this study, we consistently found that the $\gamma $ power were significantly higher in patients, which may be associated with high-risk seeking behaviors or impulsiveness deficit [6]. Taken together, patients with MUD persistently exhibit resting $\ominus $ and $\gamma $ EEG abnormalities irrelevant to drug cues, which may result from the MA-associated frontostriatal neurotoxicity (see [38] for a review).

### D. Study Contributions and Considerations

In this study, we proposed a fusion model to enhance the separation accuracy of patients with MUD from healthy subjects without the induction of drug cue reactivity. The added value of the fusion model is grounded in physiology, as MUD affects both the central and peripheral nervous systems, leading to abnormalities in EEG (central nervous systems) [6], [13], HRV, and GSR (controlled by peripheral nervous system) [8] during and after drug-cue induction. This study further confirmed that the MUD-related abnormalities in EEG, HRV and GSR exist without drug-cue induction and the combination of these modalities can improve the MUD detection rate when compared to individual modality.

Regarding the best model, the fusion of HRV and GSR achieves an accuracy of 79%, while using HRV features alone results in an accuracy of 78%. Although the improvement from adding GSR may seem modest, the value of fusion is still considered important. This is because abnormalities in HRV are not specific to MUD and can be seen in other heart-related conditions. Therefore, the proposed fusion model, which combines HRV and GSR, is likely to have a greater ability to differentiate MUD from other heart diseases, making it particularly valuable for mass screening purposes.

Previous studies have shown that MUD is associated with a range of cognitive deficits, including depression and anxiety [3], as well as the potential progression toward severe psychotic states [39], [40]. In other words, affective disorders and psychosis are common comorbidities in MUD patients. Given that this study utilizes resting-state data, it is plausible that our observed abnormalities in EEG, HRV, and GSR could be linked to these affective disorders. While our current system setup is not designed to differentiate MUD patients from individuals with other conditions like affective disorders, our system is well-suited for the primary aim of this study to facilitate mass screening for MUD. The system can effectively identify individuals without signs of MUD. For those who show positive indications of MUD, further laboratory testing is always recommended to confirm the diagnosis.

In this study, we used only five channels of EEG in this study, our classification results of fusion models are conditional on the data given. High dense EEG may provide more spatial patterns about the neuronal abnormalities in patients with MUD and result in different fusion models. Furthermore, we used a relatively small sample size for establishing the predictive model. A large-scale dataset will help clarify the findings in this study.

In conclusion, we demonstrated that a fusion model using resting-state EEG, ECG and GSR features enhances the separation accuracy of patients with MUD from healthy subjects. This fusion model is easy to implement in the clinical setting and may assist in mass screenings of methamphetamine use disorder.
